# HPLC Determination of Colistin in Human Urine Using Alkaline Mobile Phase Combined with Post-Column Derivatization: Validation Using Accuracy Profiles

**DOI:** 10.3390/molecules27113489

**Published:** 2022-05-28

**Authors:** Kalliopi Papavasileiou, Apostolia Tsiasioti, Paraskevas D. Tzanavaras, Constantinos K. Zacharis

**Affiliations:** 1 Laboratory of Analytical Chemistry, School of Chemistry, Faculty of Sciences, Aristotle University of Thessaloniki, GR-54124 Thessaloniki, Greece; papakall@chem.auth.gr (K.P.); atsiasioti@gmail.com (A.T.); ptzanava@chem.auth.gr (P.D.T.); 2 Laboratory of Pharmaceutical Analysis, Department of Pharmaceutical Technology, School of Pharmacy, Aristotle University of Thessaloniki, GR-54124 Thessaloniki, Greece

**Keywords:** Colistin A, Colistin B, high-pressure liquid chromatography, post-column derivatization, fluorescence, core–shell column, *o*-phthalaldehyde

## Abstract

In this study, the development, validation, and application of a new liquid chromatography post-column derivatization method for the determination of Colistin in human urine samples is demonstrated. Separation of Colistin was performed using a core–shell C_18_ analytical column in an alkaline medium in order (i) to be compatible with the *o*-phthalaldehyde-based post-column derivatization reaction and (ii) to obtain better retention of the analyte. The Colistin derivative was detected spectrofluorometrically (λ_ext_/λ_em_ = 340/460 nm) after post-column derivatization with *o*-phthalaldehyde and *N*-acetyl cysteine. The post-column derivatization parameters were optimized using the Box–Behnken experimental design, and the method was validated using the total error concept. The *β*-expectation tolerance intervals did not exceed the acceptance criteria of ±15%, meaning that 95% of future results would be included in the defined bias limits. The limit of detection of the method was adequate corresponding to 100 nmol·L^−1^. The mean analytical bias (expressed as relative error) in the spiking levels was suitable, being in the range of −2.8 to +2.5% for both compounds with the percentage relative standard deviation lower than 3.4% in all cases. The proposed analytical method was satisfactorily applied to the analysis of the drug in human urine samples.

## 1. Introduction

Colistin is a polypeptide with antibiotic properties, being synthesized non-ribosomally by *Bacillus polymyxa* subspecies *colistinus* [1,2]. It is a rather complex mixture consisting of structurally related compounds, with the two major ones being Colistin A (polymyxin E1) and Colistin B (polymyxin E2) [3,4,5]. Its mechanism of action against Gram-negative bacteria is based on electrostatic and hydrophobic interactions with lipopolysaccharide molecules, causing displacement of calcium and magnesium ions and destabilization of the outer membrane of organisms that are essential in antimicrobial activity [6,7]. Pharmacologically, Colistin is available in two commercial forms: Colistin sulfate is used orally and topically, while the less toxic prodrug Colistin methanesulfonate (CMS) is suitable for parenteral administration and inhalation [8].

There are two main approaches when it comes to the determination of Colistin in complicated matrices. Both include HPLC as the basic separation technique, coupled to either mass spectrometric detection or fluorescence following derivatization of the analyte through its primary amino groups. The advantages and disadvantages of these approaches in terms of cost, instrumentation, matrix interferences, and sensitivity are more or less well known, and they were adequately discussed in a recent dedicated review article by Gikas and coworkers [3]. Mass spectrometric detection techniques are always attractive in bioanalysis, and there have been several very recent reports on the analysis of Colistin in rats [9,10] or human plasma [11], intestinal matrices of poultry [12,13,14], and eggs [15]. Sample preparation prior to LC–MS analysis varies from simple protein precipitation [9] to solid-phase extraction using advanced cation exchange or hydrophilic/lipophilic balance (HLB) materials [10,14]. Typically, such methods are capable of detecting Colistin at the low ppm to ppb levels.

On the other hand, the chemical structure of Colistin contains five primary amino groups, thus being quite attractive for derivatization and fluorescence detection using a variety of suitable reagents. Nicolas et al. were the first to report the pre-column derivatization of Colistin by the well-known *o*-phthalaldehyde (OPA)/mercaptoethanol chemistry through an automated column-switching mode [16]. They also confirmed through LC–MS analyses that all five amino groups of the analyte react with OPA to form isoindole derivatives. The same chemistry was utilized—also in an automated autosampler-based derivatization scheme—to study the pharmacokinetics of inhaled Colistin [17] and in a batch mode to determine the antibiotic in medicated animal feed [18]. In the latter method, a cleaning SPE step was applied prior to derivatization. Automation of the pre-column derivatization of Colistin with OPA for feed analysis has also been reported by coupling flow injection analysis (FIA) to HPLC [19]. The FIA configuration enabled both the online automation of the ultrasound-assisted extraction of Colistin from the solid sample and the derivatization steps prior to injection and HPLC separation. Lastly, an alternative derivatizing reagent for primary amines (9-fluorenylmethyl chloroformate, FMOC) has been applied through an in-SPE mode (simultaneous SPE extraction and derivatization) for the determination of Colistin in human plasma [20,21].

In the present work, we propose an alternative to the above, the derivatization-based HPLC method for the determination of Colistin through an online post-column reaction mode (PCD). The advantages of PCD versus pre-column derivatization are well documented in the scientific literature, including simpler sample preparation and fewer matrix effects [22]. For example, protein precipitation using trichloroacetic acid (TCA)/trifluoroacetic acid (TFA) requires an extra neutralization step prior to pre-column derivatization of primary amines at alkaline pH. On the other hand, Colistin contains five primary amino groups, being susceptible to multiple reaction products if the pre-column derivatization conditions are not optimized and controlled carefully. The latter phenomenon may be intensified in complicated/unknown matrices where endogenous amino compounds may compete with the Colistin–OPA reaction. In PCD mode Colistin is derivatized reproducibly “in isolation” since the chromatographic separation of the intact analyte precedes it. Another interesting feature of our work is the employment of a core-shell analytical column that offers highly efficient chromatographic separations at highly alkaline pH values. In this way, the pH of the mobile phase (i) is perfectly compatible with the pH of the PCD reaction and (ii) improves the chromatographic behavior of the highly basic Colistin without the need for mobile phase modifiers [23]. It is quite characteristic and worth mentioning that, in [16], the authors initially attempted to develop a PCD scheme, but the reactive impurities of triethylamine (TEA) used in the mobile phase resulted in nonacceptable background fluorescence.

## 2. Results and Discussion

### 2.1. Determination of Colistin Sulfate Purity and Stoichiometry of Colistin A and B

Colistin A and B are not commercially available as individual reference standards but as a mixture (Colistin sulfate). Prior to the development and validation of the proposed HPLC–PCD method, the chromatographic purity of Colistin sulfate and the stoichiometry of Colistin A and B were evaluated. To undertake this task, an HPLC–UV system was used by employing an isocratic elution program using 10 mM borate buffer (pH 11)/ACN, 65/35 *v*/*v* on a Kinetex EVO C_18_ core–shell (100 × 4.6 mm, 2.6 μm) at a flow rate of 0.5 mL·min^−1^. The UV detection was set at 210 nm. A typical chromatogram for the purity estimation of an aqueous solution of Colistin sulfate (1000 μg·mL^−1^) is depicted in Figure S1. Looking at the elution order of the two compounds, Colistin B was eluted earlier on the reversed-phase stationary phase as it contains one fewer methylene group than Colistin A. The results (calculated using the peak normalization approach) indicated that the reference standard contained 78.6% Colistin A and 21.4% Colistin B, assuming that both components provided equal response factors. Analogous findings have been reported elsewhere [24,25].

### 2.2. Optimization of the HPLC Parameters

**Figure 1:**
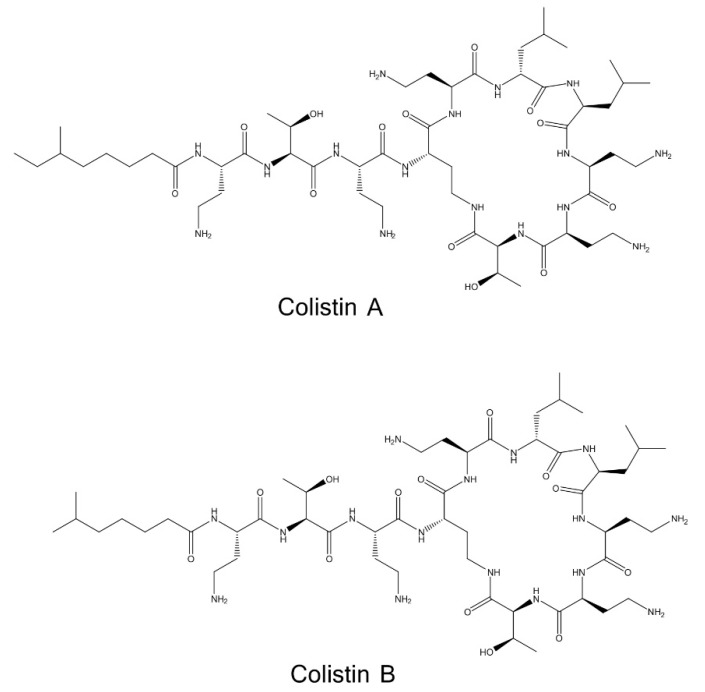
Chemical structures of Colistin A and Colistin B.

Colistin A and B are relative polar compounds and contain five unsubstituted primary amino groups in their molecules (Figure 1). According to the literature, underivatized Colistin is typically chromatographed on reversed-phase columns (e.g., C_18_) using acidic mobile phases to avoid the peak tailing by minimizing the secondary interactions of the positively charged amino groups with the uncapped silanols of the stationary phase [25,26,27]. In some cases, more complicated acidic mobile phases containing both ion-pair reagents and high concentrations of neutral salt (e.g., Na_2_SO_4_) have also been used [23]. Nevertheless, these approaches are often not attractive, especially when post-column derivatization (performed in an alkaline medium) is employed to enhance the detectability of the analyte. Such approaches typically require additional streams with high buffer concentration (e.g., 400 mM borate buffer [23]), which may lead to tubing and/or detector flow cell clogging due to reagent precipitation [22]. In our study, we investigated the chromatographic behavior of Colistin using an alkaline mobile phase to enhance the retention of the analyte on the reversed-phase column and to have better compatibility (in terms of pH) with the post-column OPA-based derivatization.

Initial experiments involved the investigation of the retention factor of Colistin in acidic and basic mobile phases. Colistin standard solution was injected separately on a core–shell C_18_ column under isocratic conditions using 20 mM phosphate buffer/ACN, 65/35, *v*/*v* at two different pH values of 3 and 11. As shown in Figure S2, an acceptable separation (R_S_ > 2.0) between Colistin A and B was observed in alkaline conditions, while both compounds were non-retained and coeluted at pH 3. In addition to phosphate buffer, we also investigated the separation efficiency using borate buffer (20 mM, pH 11). The experiments indicated that a 20% enhancement in the separation efficiency (in terms of the theoretical plates) was observed; thus, the borate buffer was adopted. In order to further improve the separation efficiency and to effectively “regenerate” the analytical column from the biological matrix, we decided to apply a simple gradient elution. Various gradient programs in combination with post-column derivatization have been tried, and a baseline-to-baseline resolution was obtained using the program described in Section 3.3.

**Figure 2:**
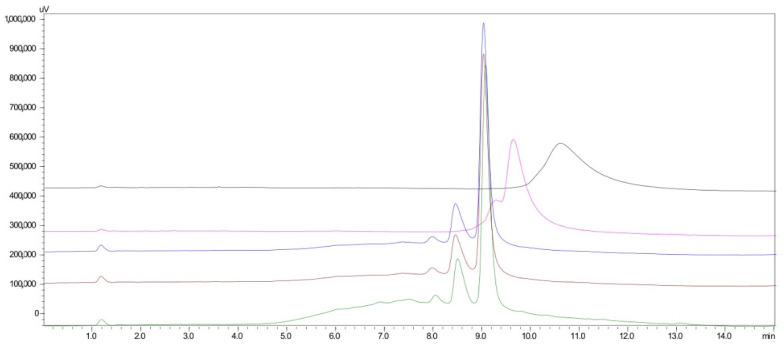
Effect of the borate buffer pH 9.5 (black line), 10.0 (fuchsia line), 10.5 (blue line), 11.0 (brown line), and 11.5 (green line) on the separation of Colistin A and B.

The pH of the mobile phase plays an important role in the separation and the peak shape by influencing the ionization of the analytes. Thus, the pH of the borate buffer was investigated at values of 9.5, 10.0, 10.5, 11.0, and 11.5. As shown in Figure 2, acceptable resolution (R_s_ > 1.5) was obtained at pH higher than 11.0. Finally, the value of 11.0 was selected for further experiments as it provided better compatibility with the pH value of the OPA reaction.

### 2.3. Optimization of the PCD Parameters Using Response Surface Methodology

The PCD experimental parameters were optimized using the response surface methodology through a Box–Behnken design (BBD). The PCD parameters consisted of the borate buffer concentration (Factor 1), the NAC concentration (C(NAC)) (Factor 2), the reaction temperature (Factor 3), the OPA amount concentration (C(OPA)) (Factor 4), and the total flow rate (Q_v_) of PCD reagents (Factor 5). The pH of the borate buffer was kept constant at a value of 11 to match the alkaline mobile phase. Preliminary experiments also confirmed the suitability of this pH for the OPA–Colistin reaction. A total of 46 experiments were conducted, and each selected factor was investigated at three levels (−1, 0, +1). The construction of the BBD and the analysis of the experimental responses were performed using the Design-Expert^®^ 13 software (Stat-Ease Inc, Minneapolis, MN, USA). The experiments were randomized to avoid the systematic error. Table S1 shows the factorial design points. For simplicity, the experiments were conducted using HPLC–PCD without the analytical column, using as carrier a mixture of 20 mM borate buffer (pH = 11.0)/ACN, 65/35% *v*/*v*. The summary of peak heights of both compounds was measured.

**Figure 3:**
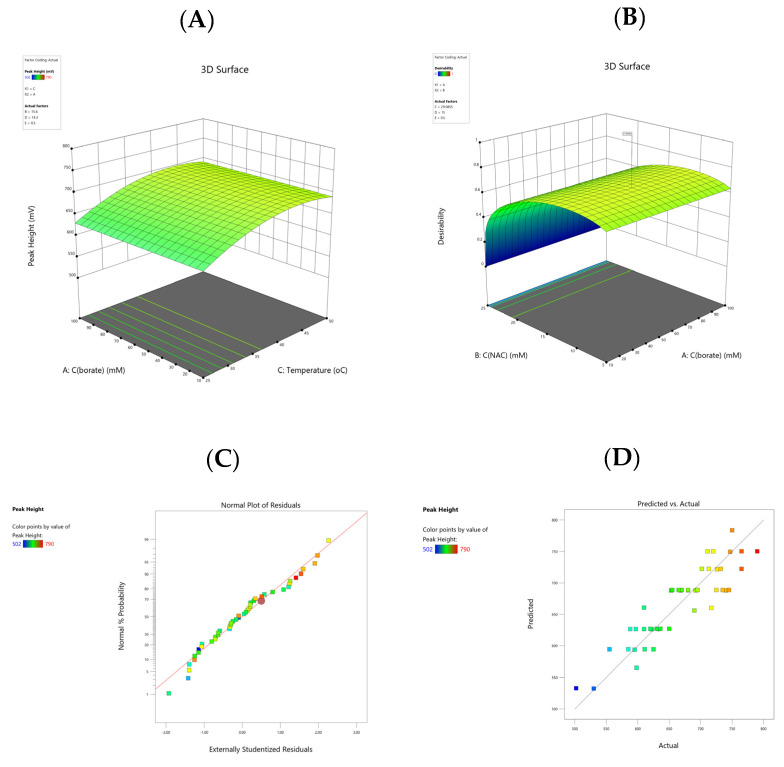
(**A**) Response surface plots indicating the effects of C (borate) and the reaction temperature on the peak height; (**B**) desirability 3D plot for defining the optimal conditions in the Colistin response; (**C**) normal plot of residuals; (**D**) predicted vs. actual values for the Colistin peak height.

The estimates of the coefficients for the models were estimated by least squares multi-linear regression, and these models were validated by analysis of variance (ANOVA) (Table 1). The nonsignificant factors (*p* > 0.05) were excluded using the “backward elimination” process. A valid ANOVA was obtained (residuals were normally distributed with constant variance) and, therefore, no transformation of the peak height was required. The adjusted and predicted *R^2^* values were 0.7880 and 0.7475, respectively, revealing that the described models have adequate reliability and predictability. The adequate precision value was 22.99, which is much higher than 4, indicating the significance of the model. The lack-of-fit (LoF) parameter (*p* = 0.8544) was obtained by the comparison of the variability of the actual model residuals with those of replicate settings and found to be nonsignificant (*p* > 0.05). As can be seen in 3D plots (Figure 3A), there was a moderate increase in Colistin response at elevated values of reaction temperature at intermediate borate buffer concentration.

Diagnostic plots such as the normal probability plot of residuals and the plot of residuals against the predicted values are illustrated in Figure 3C,D. The data were randomly scattered around the line, indicating that the model properly fit the data.

The next step involved the optimization of the Colistin response utilizing Derringer’s desirability function (D). This function was based on a scale of desirability varying between 0 and 1 as a fully desirable response and calculated by combining single desirability functions, usually as the geometric mean [28]. In our case, the goal of the optimization was set to maximize the Colistin peak height by minimizing the concentration of NAC and OPA reagents to be compliant with the requirements of green analytical chemistry including reduced consumption of hazardous organic substances [29]. The total PCD flow rate was set to 0.5 mL·min^−1^. The desirability 3D surface is depicted in Figure 3B. The optimization of D resulted in the following predicted values: borate buffer concentration = 10.52 mmol·L^−1^, C(NAC) = 11.08 mmol·L^−1^, reaction temperature = 25.08 °C, and C(OPA) = 16.14 mmol·L^−1^. For simplicity of operation, the borate buffer concentration, C(NAC), reaction temperature, and C(OPA) were rounded off to 10.5 mmol·L^−1^, 11 mmol L^−1^, 25 °C, and 16 mmol·L^−1^, respectively, and were adopted for the subsequent experiments.

### 2.4. Method Validation

**Figure 4:**
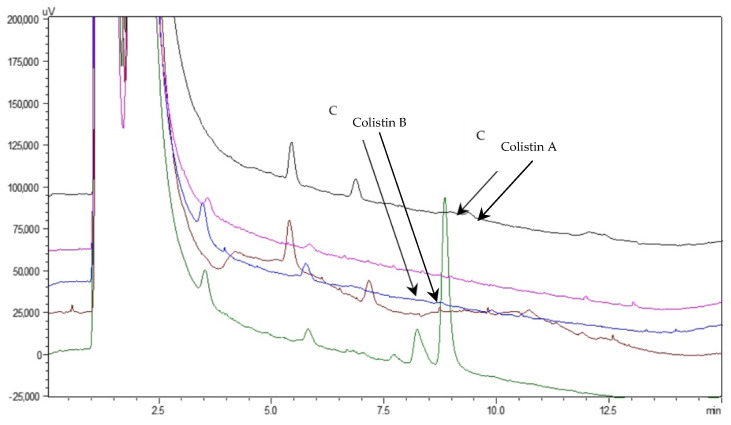
Representative HPLC–PCD chromatograms of the analysis of drug-free urine samples (brown, blue, fuchsia, and black line) and spiked with Colistin at the concentration level of 1750 nmol·L^−1^ (green line).

The developed HPLC–PCD method was validated on the basis of the accuracy profiles (for more details, see Section 3.5). The validation data for Colistin A and B are tabulated in Table 2 and Table S2 (Supplementary Materials), respectively. The selectivity of the proposed method was tested by analyzing pooled blank drug-free (*n* = 6) and spiked urine samples. Figure 4 shows a representative analysis of drug-free urine samples 10-fold diluted and spiked with Colistin standard. As derived, no interfering peaks from the biological matrix were recorded in the elution region of the Colistin A and B, revealing the adequate selectivity of the method.

**Figure 5:**
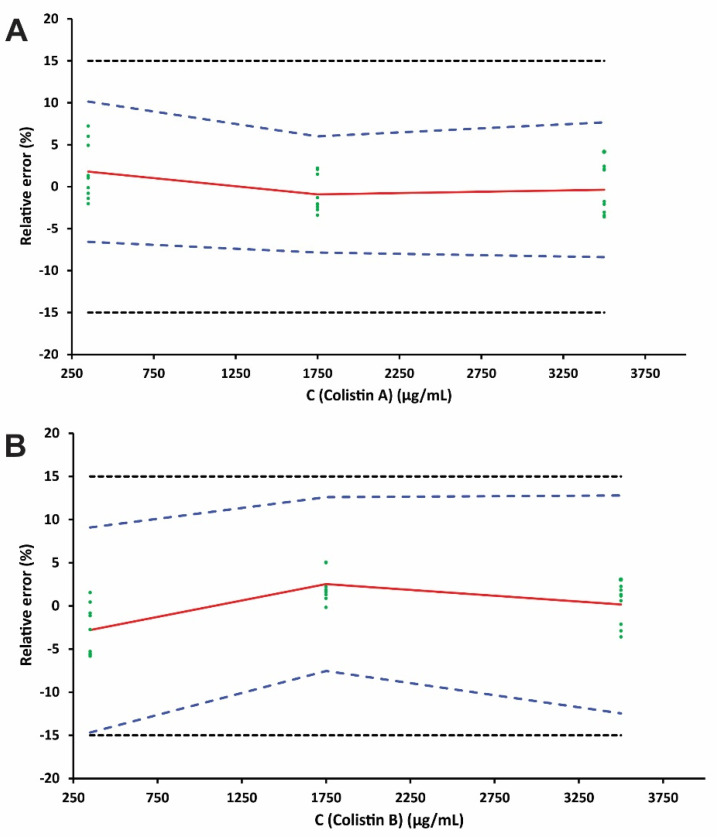
Accuracy profiles for the (**A**) Colistin A and (**B**) Colistin B determination in human urine samples. The red plain, blue dashed, and blank dotted lines represent the relative error (%), the accuracy profile, and the acceptance limits λ (±15%), respectively.

**Figure 6:**
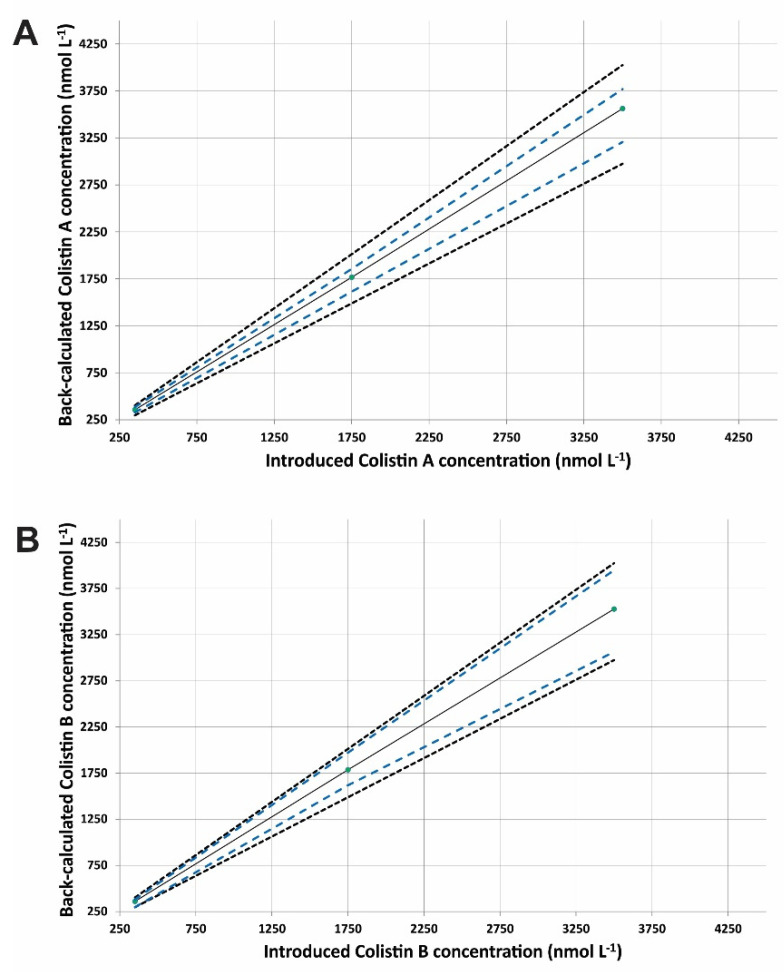
Linearity profile of (**A**) Colistin A and (**B**) Colistin B. The plain blank line corresponds to the identity line (*Y* = *X*), the blue dashed line represents the accuracy profile (*β*-ΕΤΙ), and the dotted curves illustrate the acceptance limits λ ±15% expressed in nmol·L^−1^.

The linearity of the method was investigated by selecting the most appropriate response function as the main decision criterion to eliminate the risk of future errors in the analytical method. The mean relative bias, the intermediate precision (s_r_ %), and the upper and the lower *β*-ETI were determined using the back-calculated concentrations of the validation standards for each regression model where the acceptance limits were set at ±15%. We examined only the simple unweighted regression model as it provided sufficient performance in terms of precision and accuracy. Figure 5 illustrates the fitting profiles of unweighted linear regression for both compounds. The scattering of the results was entirely inside the acceptance limits for all examined calibration levels. The linearity was also examined by fitting least-squares regression lines on the back-calculated concentrations of the validation standards against the introduced drug concentration (Figure 6). The LODs were 100 nmol·L^−1^ according to the signal-to-noise ratio criterion (S/N = 3), which are comparable to or even better than other published methods [21,30]. The LLOQ was fixed to 350 nmol·L^−1^ for Colistin A and B as derived from the accuracy profiles. The relative biases ranged between −0.4% and 1.8% for Colistin A and between −2.8% and 2.5% for Colistin B, indicating satisfactory trueness of the approach. The method precision (s_r_, %) and time-dependent intermediate precision (s_R_, %) for each concentration level were lower than 3.4% in all cases. The upper and lower *β*-ETIs for each level for both compounds were entirely included inside the acceptance limits of ±15%; therefore, the method can be considered accurate in the tested range.

The robustness of the developed PCD method was investigated using Monte Carlo simulations and capability analysis. A total of 100 iterations were conducted using Monte Carlo simulations experiments, and the simulated data were utilized to estimate the Cpk values. The acceptance criterion of the Colistin peak height was established at ±3% of the predicted value obtained from the optimization step. A set of simulation experiments were performed considering the optimum values of each parameter C(NAC), temperature, and flow rate with standard deviation (SD) values of 0.2, 0.05, and 0.01, respectively. The capability analysis resulted in a satisfactory CpK value of 1.45. Figure S3 illustrates the histogram from the capability analysis of the examined response.

### 2.5. Sample Stability

**Figure 7:**
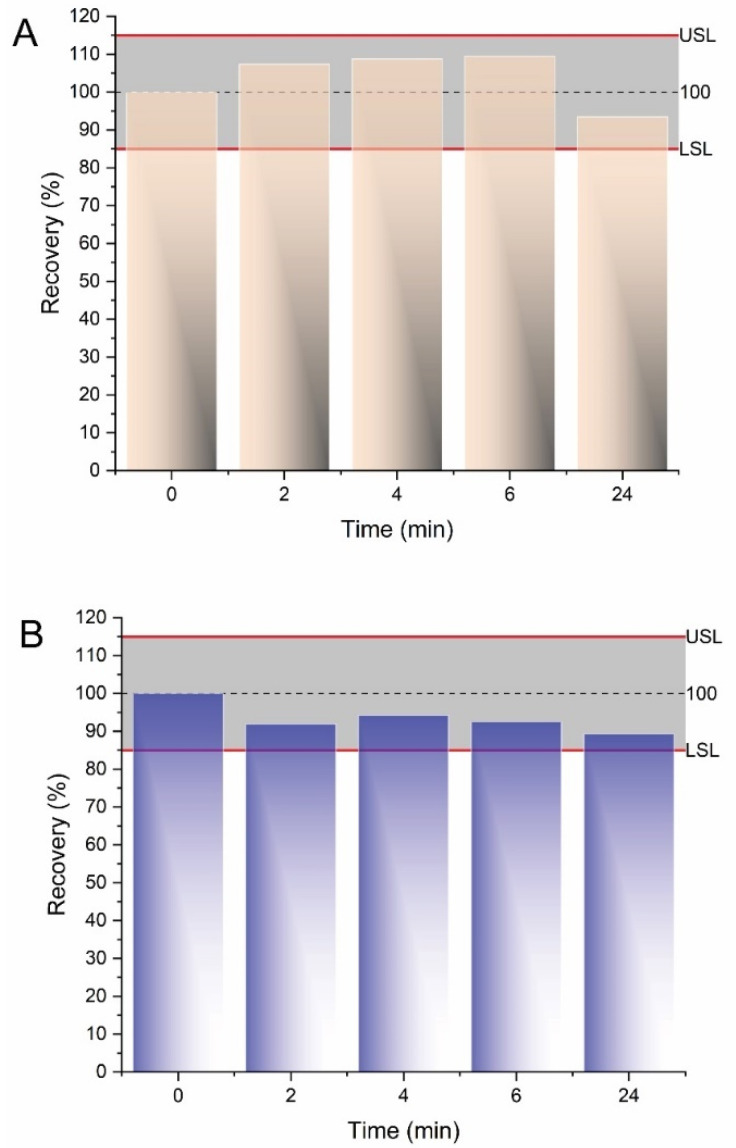
Stability data of (**A**) Colistin A and (**B**) Colistin B at ambient temperature in unprocessed urine samples. The lower specification limit (LSP) and the upper specification limit (USP) of the percentage recovery were set at 85% and 115%, respectively.

The long-term stability of Colistin A and B in urine samples was evaluated by storing unprocessed samples at ambient temperature up to 24 h and at 4 °C, as well as freeze/thaw cycle stability after three cycles at −18 °C. The experiments indicated that both analytes were stable in the period of 24 h at ambient temperature (Figure 7) as the percentage recoveries ranged between the lower specification limit (LSL) and the upper specification limit (USP) of the method accuracy. Furthermore, for the storage conditions at 4 °C and during the freeze/thaw cycles at −18 °C, the percentage recoveries ranged between 86.3% and 103.4% in all cases.

### 2.6. Sample Analysis

The applicability of the proposed HPLC–PCD method was further evaluated by analyzing human urine samples by spiking the samples at different concentration levels in the range of 350–3500 nmol·L^−1^. All samples were treated according to the procedure described in Section 3.4. The average recoveries for all analytes ranged from 86.7% to 114.1%, while the relative standard deviation was lower than 3.2%, showing the good recovery and absence of matrix effects (Table 3). The proposed method is feasible for quantitative analysis of Colistin A and B in urine samples and, therefore, could be used in routine analysis.

## 3. Materials and Methods

### 3.1. Reagents and Solutions

Colistin disulfate (potency > 77%) was provided by AppliChem GmbH (Darmstadt, Germany). Potassium hydrogen phosphate, sodium hydroxide (pellets), boric acid, *N*-acetyl cysteine (NAC), and *o*-phthalaldehyde (OPA) (>97%) were of analytical grade and purchased by Sigma-Aldrich (St. Louis, Mo, USA). HPLC-grade acetonitrile (ACN) and methanol (MeOH) were obtained from Honeywell (Morris Plains, NJ, USA). Other reagents were of analytical grade and were purchased from Merck (Merck KGaA, Darmstadt, Germany). A B30 water purification system (Adrona SIA, Riga, Latvia) was used for the production of Milli-Q water (<0.2 μS/cm at 25 °C).

Stock standard solution of Colistin sulfate (865 μmol·L^−1^) was prepared by dissolving the appropriate amount in 10 mL of water. This solution was kept at 4 °C for 10 days. Working solutions of Colistin were prepared by serial dilution from the stock solution in the same diluent. An OPA solution at a concentration of 16 mmol·L^−1^ was prepared by dissolving the appropriate amount in 5 mL of methanol followed by dilution to 100 mL with Milli-Q water. *N*-Acetyl cysteine (NAC) solution was prepared at an amount concentration of 11 mmol·L^−1^ in 10.5 mmol·L^−1^ borate buffer (pH = 11.0). The PCD reagent solutions were stable for at least three working days when stored refrigerated in an amber glass container.

Phosphate or borate buffers were prepared and were adjusted to the desired pH value via dropwise addition of 1.0 mol·L^−1^ NaOH or H_3_PO_4_ solution. The mobile phase was filtrated under vacuum through 0.45 μm membrane filters (Whatman^®^).

### 3.2. HPLC Instrumentation

Chromatographic analyses were performed using a Shimadzu HPLC–FLD setup (Kyoto, Japan) consisting of two LC-20AD high-pressure gradient pumps, a SIL-20AC autosampler a CTO-10ASVP column compartment, and an RF-535 fluorescence detector. For the determination of Colistin purity and stoichiometry, the above system was coupled to an SPD-M20A photodiode array detector. All separations were performed on a Kinetex EVO C_18_ core–shell (100 × 4.6 mm, 2.6 μm). The control of the instrument and the data acquisition were carried out via the LC Solutions software (vs 1.25 SP4).

The PCD instrumentation involved a Minipuls^TM^ 3 peristaltic pump (Gilson, UK), a PTFE reaction coil (200 cm × 0.5 mm i.d.), a reaction coil thermostat (HiChrom Limited, Burbank, CA, USA), and a TEE-connector (Upchurch Scientific^®^).

### 3.3. HPLC–PCD Conditions

The mobile phases A and B consisted of aqueous borate buffer (20 mmol·L^−1^, pH = 11) and ACN, respectively. The analyte was separated from the endogenous compounds of the urine matrix using a binary gradient elution program starting from 20% *v*/*v* B and linearly increased to 60% in 10 min, before returning to the initial concentration (20% *v*/*v*) at 13 min and holding for 25 min to obtain reproducible separations. The column temperature was kept at 25 °C. The injection volume was 20 μL, while the flow rate of the mobile phase was 1 mL·min^−1^. After separation, the analyte was reacted online downstream with a buffered OPA reagent. A borate buffer/NAC solution (10.5 mmol·L^−1^, pH = 11, C(NAC) = 11 mmol·L^−1^) was premixed with an OPA solution (16 mmol·L^−1^) channel at equal flow rate (0.25 mL·min^−1^ each), and the resulted solution was merged with the mobile phase stream. The Colistin–OPA derivatives were detected spectrofluorometrically at λ_ext_ = 340 nm and λ_em_ = 460 nm. Between injections, the autosampler was rinsed with 500 μL of water/methanol, 50/50% *v*/*v* to avoid potential carry-over.

### 3.4. Sample Preparation

Urine samples were collected from healthy volunteers in sterilized containers and kept at −18 °C until their analysis. No medication was received prior to the sample collection. The samples were centrifuged and diluted 10-fold before HPLC–PCD analysis.

For method validation, calibration standards at three concentration levels (*m* = 3), namely, 350, 1750, and 3500 nmol·L^−1^, were prepared in triplicate (*n* = 3) for each series of experiments (*k* = 3). The validation standards comprised urine spiked with known amounts of Colistin which were prepared from independent stock solutions. Three series of standards were prepared by spiking the exact volume of the stock solutions of the analyte to obtain the desired concentration level.

### 3.5. Method Validation Using Accuracy Profiles

The validation of the proposed analytical method was performed by taking into consideration the total error (systematic and random errors) as per “Société Française des Sciences et Techniques Pharmaceutiques” (SFSTP) harmonization guidelines [31]. This approach includes the construction of the accuracy profiles which is a decision-making graphical tool that helps the analyst to decide about the validity of the analytical method. The theory behind this concept lies on the fact that the result X concentration obtained from an analytical procedure is different from the unknown “true value” μ of the analyzed sample and is expected to be less than an acceptable limit λ.

−λ < Χ − μ < λ or |X − μ| < λ.

The acceptable limit λ depends on the objectives of the analytical method and is typically expressed as the target value. For example, λ can be 15% in bioanalysis. The selection of the suitable response function (e.g., linear, with and without transformation, weighted, unweighted) was based on the accuracy profiles and the *β*-expectation tolerance interval (*β*-ΕΤΙ). The latter parameter is the interval where it is expected that a proportion *β* of future measurements would be within the acceptance limits ±λ. The expression of the analytical profile is given below.

$$\left[bias{\left(\%\right)}_{j}-Q_{t}\left(v;\frac{1+\beta }{2}\right)\sqrt{1+\frac{1}{pnB_{j}^{2}}s_{r,j}\;};bias{\left(\%\right)}_{j}+Q_{t}\left(v;\frac{1+\beta }{2}\right)\sqrt{1+\frac{1}{pnB_{j}^{2}}s_{r,j}\;}\right],$$

where *bias*(%)*_j_* = $\frac{\mu_{j}-\mu_{Tj}}{\mu_{Tj}}\times 100$, *s_r,j_* = $\frac{{\hat{\sigma }}_{W,j}^{2}+{\hat{\sigma }}_{B,j}^{2}}{\mu_{j}}\times 100$, *B_j_* =$\sqrt{\frac{\frac{{\hat{\sigma }}_{Β,j}^{2}}{{\hat{\sigma }}_{W,j}^{2}}+1}{n\frac{{\hat{\sigma }}_{Β,j}^{2}}{{\hat{\sigma }}_{W,j}^{2}}+1}}$, ν = $\frac{{\left(R+1\right)}^{2}}{R+\frac{1}{n\left(p-1\right)}+1-\frac{1}{pn^{2}}}.$

– $\;\mu_{j}$ is the estimate of the mean results of the *j-*th concentration level,
– $\;\mu_{T}$ is the unknown “true value”,
– *p* is the number of series,
– *n* is the number of the independent replicate per series,
– *Q_t_* (*ν*; (1 + *β*)/2) is the *β* quantile of the *t*-Student distribution with *ν* degrees of freedom,
– $\;{\hat{\sigma }}_{W,j}^{2}$ is the within-series variance,
– ${\hat{\sigma }}_{B,j}^{2}$ is the between-series variance.

## 4. Conclusions

In this research, an HPLC–PCD method was developed and validated for the determination of Colistin in human urine. The proposed analytical scheme offers some interesting features that are summarized as follows: (i) from a chromatographic point of view, a novel core–shell column compatible with highly alkaline mobile phases enabled the separation of the analyte using a simple mobile phase, avoiding modifiers; (ii) the pH of the mobile phase was perfectly matched with the alkaline pH of the derivatization reaction; (iii) post-column derivatization of the “isolated” Colistin isomers offered the potential to analyze biological material following minimal sample preparation; (iv) adequate sensitivity at the low micromolar levels was achieved.

## Figures and Tables

**Table 1:** Analysis of variance (ANOVA) for Box–Behnken design.

Source	Sum of Squares	Df ^1^	Mean Square	F-Value		*p*-Value
Model	1.578 × 10^5^	5	31,567.80	34.45	<0.0001	significant
B-C(NAC)	65,280.25	1	65,280.25	71.24	<0.0001	
C-Temperature	15,006.25	1	15,006.25	16.38	0.0002	
E-Qv (PCD) (Total)	60,639.06	1	60,639.06	66.17	<0.0001	
B^2^	9409.42	1	9409.42	10.27	0.0027	
C^2^	10,039.22	1	10,039.22	10.96	0.0020	
Residual	36,655.39	40	916.38			
LoF	29,272.06	35	836.34	0.5664	0.8544	not significant
Pure Error	7383.33	5	1476.67			
Cor Total	1.945 × 10^5^	45				

^1^ Degrees of freedom.

**Table 2:** Validation results for the quantitation of Colistin A in a pooled drug-free urine sample (*n* = 6).

Validation Criteria			
Response Function (Linear Unweighted)	Slope	Intercept	*r*
(*k* ^a^ = 3; *m* = 3; *n* = 3) (350–3500 nmol·L^−1^)			
Day 1	307.96	−55,640	0.9985
Day 2	353.28	−86,169	0.9997
Day 3	345.59	−80,842	0.9967
Precision (*k* = 3; *n* = 3)			
C (nmol·L^−1^)	*s*_r_ (%) ^b^	*s*_R_ (%) ^c^	
350	3.4	3.4	
1750	1.7	2.3	
3500	2.7	3.1	
Trueness (*k* = 3; *n* = 3)			
C (nmol·L^−1^)	Relative bias (%)		
350	1.8		
1750	−0.9		
3500	−0.4		
Accuracy (*k* = 3; *n* = 3)			
C (nmol·L^−1^)	Relative *β*-ΕΤΙ (%)		
350	[−6.57, 10.15]		
1750	[−7.84, 6.0]		
3500	[−8.39, 7.66]		
Linearity (*k* = 3; *n* = 3; *m* = 3) (350–3500 nmol·L^−1^)			
Slope	1.018		
Intercept	−5.74		
*r* ^2^	0.9999		
LOD (nmol·L^−1^)	100		
LLOQ (nmol·L^−1^)	350		

^a^ *k*, *m*, and *n* correspond to the number of experiments, calibration levels, and replicates, respectively. ^b^ *s*_r_ (%): relative standard deviation under repeatability conditions. ^c^ *s*_R_ (%): relative standard deviation under intermediate precision.

**Table 3:** Analysis of representative urine samples using the proposed HPLC–PCD method.

Sample	Colistin A			Colistin B		
	Fortification Level (nmol·L^−1^)	Recovery (%)	RSD (%)	Fortification Level (nmol·L^−1^)	Recovery (%)	RSD (%)
Urine S1	865	108.1	1.3	865	111.4	1.6
	2580	103.9	1.7	2580	114.1	1.1
Urine S2	865	87.0	2.7	865	105.7	2.1
	2580	106.4	1.3	2580	100.9	1.7
Urine S3	865	86.7	1.2	865	108.3	2.0
	2580	107.2	1.6	2580	100.6	3.2

## Data Availability

Not applicable.

## Supplementary Materials

The following supporting information can be downloaded at https://www.mdpi.com/article/10.3390/molecules27113489/s1: Figure S1. Representative HPLC–UV chromatogram of the purity determination of Colistin reference standard (provided by AppliChem). Experimental conditions: Kinetex EVO C18 core–shell (100 × 4.6 mm, 2.6 μm), 10 mM borate buffer (pH 11.0)/ACN, 65/35 *v*/*v*, λ = 210 nm, injection volume: 10 μL, column temperature: 25 °C; Figure S2. Analysis of Colistin sulfate using 20 mM phosphate buffer/ACN, 65/35% *v*/*v*, at pH 3 (black line), and pH 11 (blue line); Figure S3. Probabilistic distribution of the peak height of Colistin during Monte Carlo simulation experiments; Table S1. Experimental runs generated by Box–Behnken design; Table S2. Validation results for the quantitation of Colistin B in pooled drug-free urine sample (*n* = 6).

## Sample Availability

Samples of the compounds are available from the authors.
