# Supplementary material for: HPLC Determination of Colistin in Human Urine Using Alkaline Mobile Phase Combined with Post-Column Derivatization: Validation Using Accuracy Profiles
Source: Molecules. 2022 May 28;27(11):3489. doi: 10.3390/molecules27113489 (PMC9181871; doi:10.3390/molecules27113489)
Supplement: Supplementary file 1 [file molecules-27-03489-s001.zip › molecules-1721544-supplementary.pdf]

molecules

MDPI

SUPPLEMENTARY MATERIAL

HPLC Determination of Colistin in Human Urine Using Alkaline Mobile Phase Combined with Post-Column Derivatization: Validation Using Accuracy Profiles

Kalliopi Papavasileiou [1], Apostolia Tsiasioti [1], Paraskevas D. Tzanavaras [1] and Constantinos K. Zacharis [2,*]

[1] Laboratory of Analytical Chemistry, School of Chemistry, Faculty of Sciences, Aristotle University of
Thessaloniki, GR-54124 Thessaloniki, Greece; papakall@chem.auth.gr (K.P.); atsiasioti@gmail.com (A.T.); ptzanava@chem.auth.gr (P.D.T.)

[2] Laboratory of Pharmaceutical Analysis, Department of Pharmaceutical Technology, School of Pharmacy, Aristotle University of Thessaloniki, GR-54124 Thessaloniki, Greece

* Correspondence: czacharis@pharm.auth.gr; Tel.: +30-2310997663

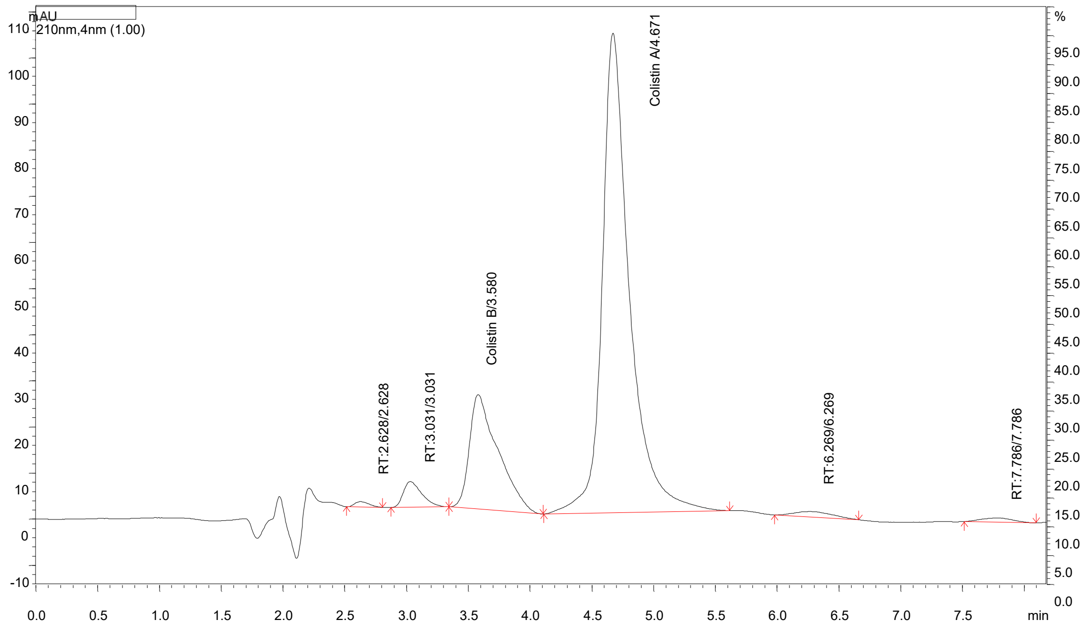

Figure S1. Representative HPLC-UV chromatogram of the purity determination of colistin reference standard (provided by AppliChem). Experimental conditions: Kinetex EVO C18 core-shell (100 × 4.6 mm, 2.6 μm), 10 mM borate buffer (pH 11.0)/ACN, 65/35 v/v, λ = 210 nm, injection volume: 10 μL, Column temperature: 25 °C.

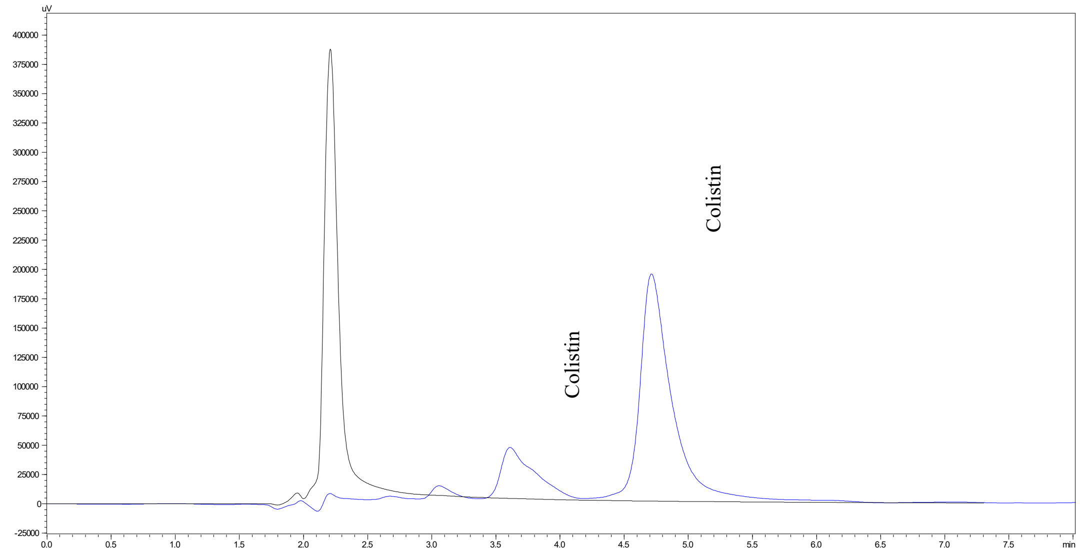

Figure S2. Analysis of colistin sulfate using 20 mM phosphate buffer/ACN, 65/35% v/v, at pH 3 (black line) and pH 11 (blue line).

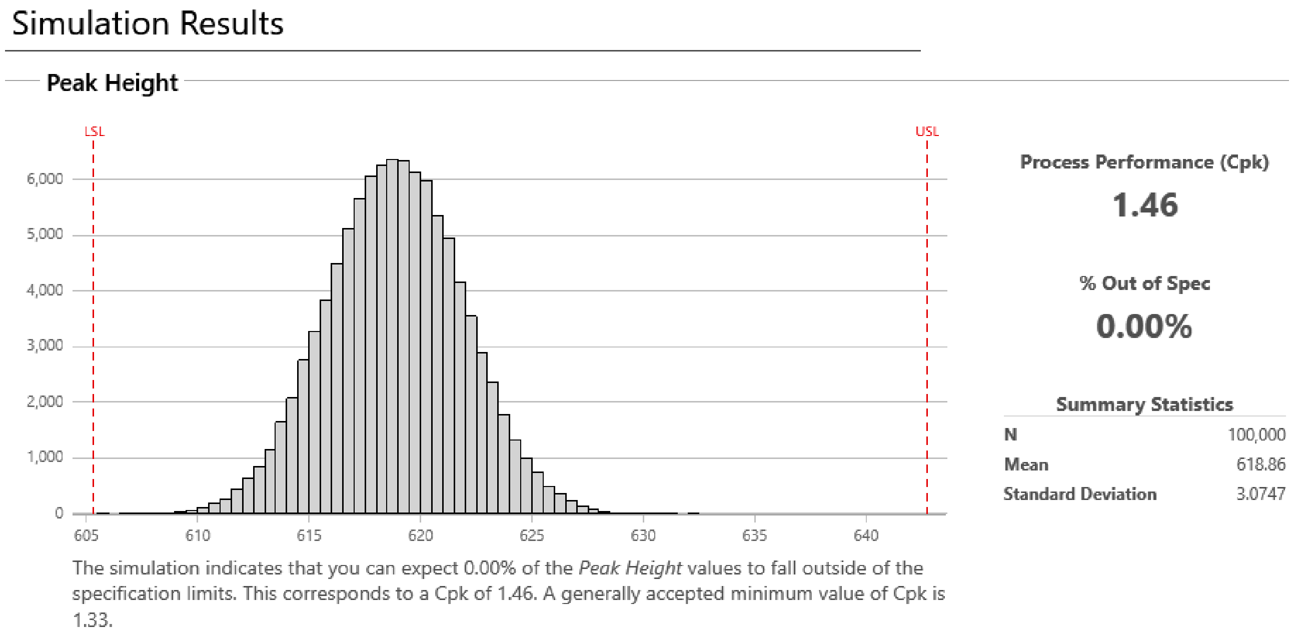

Figure S3. Probabilistic distribution of the peak height of Colistin during Monte-Carlo simulation experiments.

Table S1. Experimental runs generated by Box-Behnken design.

Run No	Borate buffer (Factor 1)	C(NAC) (Factor 2)	Reaction temperature (Factor 3)	C(OPA) (Factor 4)	Qv (PCD) (Factor 5)	Peak Height (mV)
1	10	15	50	15	0.5	740
2	10	15	37.5	15	0.25	720
3	100	25	37.5	15	0.5	713
4	55	15	37.5	15	0.5	665
5	100	15	50	15	0.5	653
6	100	15	25	15	0.5	597
7	10	15	37.5	15	0.75	620
8	55	15	50	12.5	0.5	692
9	55	15	25	15	0.75	598
10	100	15	37.5	5	0.5	695
11	55	5	37.5	15	0.75	502
12	55	25	37.5	15	0.25	750
13	55	15	37.5	15	0.5	670
14	55	25	25	15	0.5	717
15	55	25	37.5	15	0.75	610
16	55	5	25	15	0.5	530
17	55	15	37.5	15	0.5	655
18	10	15	25	15	0.5	610
19	55	25	37.5	5	0.5	702
20	55	15	37.5	12.5	0.75	636
21	55	15	37.5	15	0.5	735
22	55	15	37.5	15	0.5	745
23	55	15	50	15	0.75	622
24	55	5	37.5	12.5	0.5	625
25	100	5	37.5	15	0.5	585
26	55	15	37.5	12.5	0.25	765
27	55	5	50	15	0.5	595
28	55	5	37.5	5	0.5	555
29	55	15	25	5	0.5	588
30	10	5	37.5	15	0.5	611
31	100	15	37.5	12.5	0.5	670
32	55	15	25	15	0.25	680
33	55	25	37.5	12.5	0.5	732
34	55	5	37.5	15	0.25	690
35	100	15	37.5	15	0.75	630
36	55	15	37.5	15	0.5	680
37	100	15	37.5	15	0.25	790
38	10	15	37.5	12.5	0.5	725
39	10	15	37.5	5	0.5	695
40	55	15	50	5	0.5	668
41	55	25	50	15	0.5	726
42	55	15	37.5	5	0.25	711
43	55	15	37.5	5	0.75	650
44	44	55	15	25	12.5	633
45	45	55	15	50	15	747
46	46	10	25	37.5	15	765

Table S2. Validation results for the quantitation of Colistin B in a pooled drug-free urine sample (n = 6).

**Validation criteria**			
Response function (linear unweighted)	Slope	Intercept	r
(k^a = 3; m = 3; n = 3) (350—3500 nmol L^{-1})			
Day 1	293.46	−38168	0.9973
Day 2	331.94	−14208	0.9999
Day 3	330.24	−8400	0.9999
Precision (k = 3; n = 3)			
C (nmol L^{-1})	s_r (%)[b]	s_R (%)[c]	
350	1.6	3.2	
1750	0.8	2.6	
3500	1.8	2.8	
Trueness (k = 3; n = 3)			
C (nmol L^{-1})	Relative bias (%)		
350	−2.8		
1750	2.5		
3500	−0.2		
Accuracy (k = 3; n = 3)			
C (nmol L^{-1})	Relative β-ETI (%)		
350	[−14.68, 9.1]		
1750	[−7.55, 12.6]		
3500	[−12.46, 12.80]		
Linearity (k = 3; n = 3; m = 3) (350 – 3500 nmol L^{-1})			
Slope	1.005		
Intercept	14.48		
r^2	0.9999		
LOD (nmol L^{-1})	100		
LLOQ (nmol L^{-1})	350		

[a] k, m and n correspond to the number of experiments, calibration levels and replicates, respectively.

[b] s_r (%): relative standard deviation under repeatability conditions.

[c] s_R (%): relative standard deviation under intermediate precision.
